# The hypoxia-responsive lncRNA *NDRG-OT1* promotes NDRG1 degradation via ubiquitin-mediated proteolysis in breast cancer cells

**DOI:** 10.18632/oncotarget.23732

**Published:** 2017-12-28

**Authors:** Hsin-Chen Lin, Ching-Ching Yeh, Lo-Yun Chao, Mong-Hsun Tsai, Hung-Hsin Chen, Eric Y. Chuang, Liang-Chuan Lai

**Affiliations:** ^1^ Graduate Institute of Physiology, National Taiwan University, Taipei, Taiwan; ^2^ Institute of Biotechnology, National Taiwan University, Taipei, Taiwan; ^3^ Bioinformatics and Biostatistics Core, Center of Genomic Medicine, National Taiwan University, Taipei, Taiwan; ^4^ Graduate Institute of Biomedical Electronics and Bioinformatics, National Taiwan University, Taipei, Taiwan

**Keywords:** hypoxia, lncRNA, NDRG1-OT1, NDRG1, ubiquitination

## Abstract

Hypoxia can lead to solid tumor aggressiveness by driving multiple signaling pathways. Long non-coding RNAs respond to several extrinsic stimuli, causing changes in cancer cells by participating in multiple steps of gene expression. However, genomic profiling of long non-coding RNAs regulated by oxygen in breast cancer remained unclear. Therefore, the aims of this study were to identify oxygen-responsive long non-coding RNAs in breast cancer cells, and to delineate their regulatory mechanisms. The expression profiling of long non-coding RNAs in breast cancer cells growing under normoxic, hypoxic, and re-oxygenated conditions was examined using next-generation sequencing technology. Four hundred and seventy-two lncRNAs oxygen-responsive lncRNAs were identified. After examining the top three differentially expressed lncRNAs in hypoxia, we selected N-Myc Downstream Regulated Gene 1-Overlapping 1 (*NDRG1-OT1*) for further study, especially the most responsive isoform, *NDRG1-OT1*_v4. We overexpressed *NDRG1-OT1*_v4 under normoxia and performed microarray analysis to identify 108 *NDRG1-OT1*_v4 regulated genes and their functions. Among these genes, we found that both *NDRG1* mRNA expression and NDRG1 protein levels were inhibited by *NDRG1-OT1*_v4. Finally, we used co-immunoprecipitation to show that *NDRG1-OT1*_v4 destabilizes NDRG1 by promoting ubiquitin-mediated proteolysis. Our findings reveal a new type of epigenetic regulation of *NDRG1* by *NDRG1-OT1*_v4 in breast cancer cells.

## INTRODUCTION

Hypoxia is a crucial determinant of the aggressiveness of solid tumors [1]. Because of the rapid growth of tumor cells and inadequate vascular distribution in the tumor microenvironment, an imbalance in oxygen delivery and consumption results in hypoxia [2, 3]. Previous studies have shown that hypoxia can affect the proliferation, invasion, and metastasis of tumors [4, 5], the induction of apoptosis [6], and angiogenesis [7]. Tumors become more aggressive as a result.

N-myc downstream-regulated gene 1 (*NDRG1*) has various effects on tumor cell function. It has been reported to respond to many stressful environments, such as DNA damage [8] and hypoxia [9, 10]. In hypoxia, up-regulation of *NDRG1* leads to cellular differentiation, proliferation, re-distribution of the cell cycle, and metastasis [11–13]. The expression of *NDRG1* depends on both transcriptional and epigenetic regulation. In transcriptional regulation, many transcription factors, such as HIF-1 [13], aryl hydrocarbon receptor (AHR) [14], and activating protein 1 (AP-1) [15], have been reported to bind to *NDRG1*'s promoter and activate its expression. Regarding epigenetic regulation, *NDRG1* was reported to be regulated epigenetically by histone acetylation [16] and microRNA-769-3p [17]. However, no studies have indicated that *NDRG1* is regulated by DNA methylation or non-coding RNA.

Long non-coding RNAs (lncRNAs) comprise a subgroup of non-coding RNAs whose lengths are greater than 200 nucleotides. Recent studies have indicated that many lncRNAs respond to cellular stresses, such as drugs [18], hormones [19, 20], radiation [21, 22], and hypoxia [23–25]. lncRNAs respond to these stimuli by participating in several steps of gene expression, leading to changes in cellular function. For example, lncRNAs epigenetically regulate gene expression [26, 27], inhibit target gene degradation [28], block the effect of microRNA (miRNA) by forming a sponge structure to absorb miRNA [29, 30], and regulate synthesis and degradation of target proteins [31, 32]. Although some oxygen-responsive lncRNAs have been identified and play a role in the onset of several cancers [27, 32], genomic profiling of lncRNAs regulated by oxygen in breast cancer remained to be explored.

In this study, next-generation sequencing was used to identify oxygen-responsive lncRNAs in MCF-7 breast cancer cells. Among these differentially expressed lncRNAs, we chose a hypoxia-induced lncRNA, *NDRG1-OT1*, for further analysis, particularly the isoform *NDRG1-OT1*_v4, which had the highest expression levels in hypoxia. In order to identify genes regulated by *NDRG1-OT1*_v4, the genomic expression profiling of MCF-7 cells overexpressing *NDRG1-OT1*_v4 in normoxia was examined. Among the *NDRG1-OT1_v4*-regulated genes, we focused on *NDRG1* and found that *NDRG1-OT1*_v4 inhibited NDRG1 expression by promoting NDRG1 degradation via the ubiquitin-proteasome pathway.

## RESULTS

### Identification of lncRNA expression profiles in different O_2_ conditions

**Figure 1:**
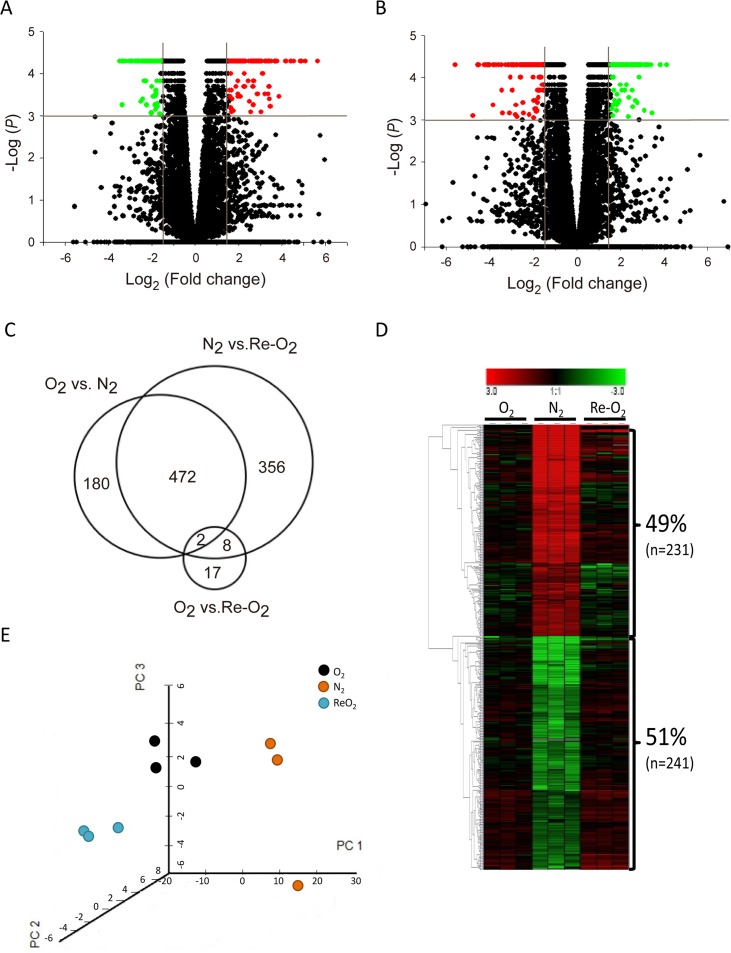
Identification of oxygen-responsive lncRNAs in MCF-7 cells using next generation sequencing analysis **(A)** Volcano plots of differentially expressed lncRNAs responding to hypoxia (N_2_). Criteria for selecting O_2_-responsive lncRNAs: fold change >3X and *P* <0.001. Red points: up-regulated lncRNAs in N_2_; green points: down-regulated lncRNAs in N_2_. **(B)** Volcano plots of differentially expressed lncRNAs responding to reoxygenation (ReO_2_). Criteria for selection were as in panel (A). Red points: down-regulated lncRNAs in ReO_2_; green points: up-regulated lncRNAs in ReO_2_. **(C)** Venn diagram of differentially expressed lncRNAs in different O_2_ conditions. O_2_: normoxia. **(D)** Heat map and hierarchical cluster analysis of O_2_-responsive lncRNAs. Red: up-regulated lncRNAs in N_2_ and down-regulated in ReO_2_; green: down-regulated lncRNAs in N_2_ and up-regulated in ReO_2_. **(E)** Principal component analysis (PCA) of O_2_-responsive lncRNAs. PCA was plotted using expression of differentially expressed probes after quantile normalization. Each dot represents each sample. Three independent experiments were done in normoxia (black), hypoxia (orange), and re-oxygenation (cyan) respectively.

In order to examine all lncRNAs regulated by O_2_ in MCF-7 cells, cells were harvested under normoxia (O_2_), hypoxia (N_2_), and re-oxygenation (Re-O_2_) conditions in triplicate. The lncRNA expression profiles were analyzed using a next-generation sequencing (NGS) system. A Student's t-test was conducted to examine the difference in FPKMs between each condition. The criteria for selecting O_2_-responsive lncRNAs consisted of fold change > 3 and significant differences (*P* <0.001) between the N_2_ and O_2_ conditions (Figure 1A) as well as Re-O_2_ and N_2_ (Figure 1B). In addition, lncRNAs were further filtered to include only those with an opposite response between shifting from normoxia to hypoxia and from hypoxia to re-oxygenation, but similar expression levels between normoxia and re-oxygenation (Figure 1C). In total, 472 lncRNAs met these criteria (Figure 1C). Among them, 49% (n = 231) were up-regulated in N_2_ and down-regulated in Re-O_2_, while 51% (n = 241) were down-regulated in N_2_ and up-regulated in Re-O_2_ (Figure 1D). Lastly, principal component analysis (PCA) using expression levels of these 472 O_2_-responsive lncRNAs also showed distinct clusters of transcriptional responses for each condition (Figure 1E).

### *NDRG1-OT1* was hypoxia responsive lncRNA

**Figure 2:**
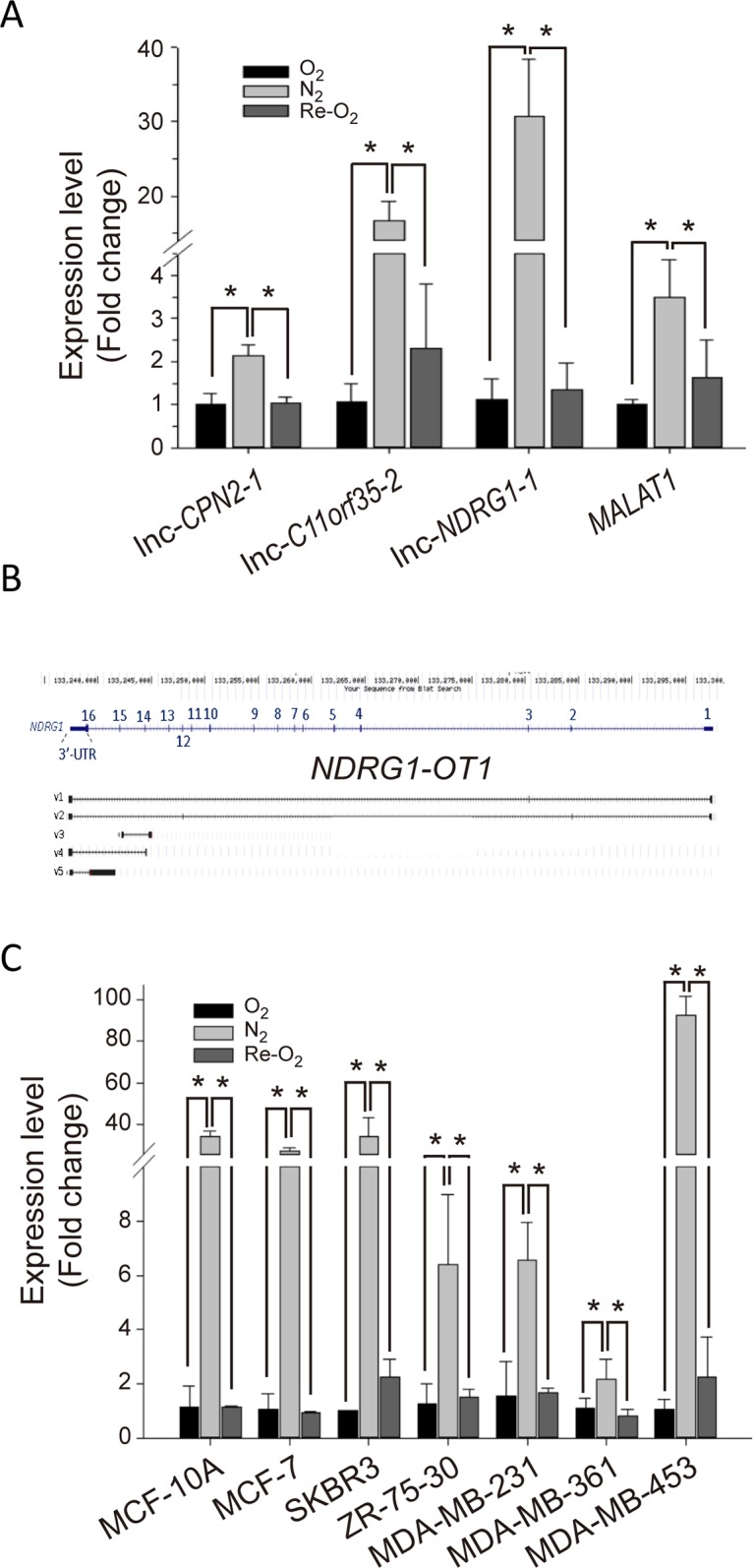
*NDRG1-OT1*_v4 is up-regulated in hypoxia and down-regulated in re-oxygenation **(A)** Validation of *lnc-CPN2-1*, *lnc-C11orf35-2*, *lnc-NDRG1-1* and *MALAT1* expression levels among different O_2_ conditions by quantitative RT-PCR. Internal control: 18s. Positive control for hypoxia: *MALAT1* [33]. The relative expression level of each condition was normalized to O_2_. Data were repeated at least 3 times, and the results are means ± SDs. ^*^, *P* <0.05. **(B)** Schematic diagram of *NDRG1-OT1* isoforms. Transcription of *NDRG1-OT1* was overlapped with *NDRG1*, located in chromosome 8. The exon number and position are shown on the sequence of *NDRG1*. v1-v5 indicate the different isoforms. **(C)** Expression profiling of *NDRG1-OT1*_v4 in six breast cancer cells and one breast normal cell line (MCF-10A) under different O_2_ conditions.

Among these differentially expressed lncRNAs, we used quantitative RT-PCR to validate the expression profiles of the top three hypoxia-inducible lncRNAs: lnc-*CPN2-1*, lnc*-C11orf35-2* and lnc-*NDRG1-1*. LncRNA *MALAT1* was used as a positive control for hypoxia [33]. Similar to the expression pattern in NGS, all three lncRNAs were significantly up-regulated under N_2_ and down-regulated under Re-O_2_ (Figure 2A). Especially, we were particularly interested in lnc-*NDRG1-1*. The main reason was that lnc-*NDRG1-1* was located in *NDRG1* (Figure 2B), which has been systemically investigated in our lab [14, 17, 34]. Therefore, we decided to investigate the relationship between this lncRNA and *NDRG1* in the following experiments. In addition, following the guidelines on gene nomenclature of the Human Genome Organization (HUGO), we renamed lnc-*NDRG1-1* as *NDRG1-OT1*, which it is called hereafter. As shown in Figure 2B, *NDRG1-OT1* has five isoforms. The expression levels of these isoforms in MCF-7 cells under different O_2_ conditions were different (Table 1). Among them, excluding v1 and v3 transcripts that did not express in normoxia, the v4 transcript had the highest expression level in N_2_ and the largest fold changes among different O_2_ environments. The phenomenon were observed in other breast cancer cell lines (Figure 2C). Therefore, we investigated the role of this isoform in the regulatory mechanisms of *NDRG1-OT1* on its target genes under different O_2_ conditions.

**Table 1:** The amount of *NDRG1-OT1* isoforms among different O_2_ conditions

*NDRG1-OT1 isoform*	O_2_		N_2_		ReO_2_	
	FPKM (%)	Read count	FPKM (%)	Read count	FPKM (%)	Read count
*NDRG1-OT1_v1*	0.0 (0.0)	0.0	0.0 (0.0)	0.0	0.0 (0.0)	0.0
*NDRG1-OT1_v2*	4.6 (53.4)	16.3	119.7 (38.1)	441.0	2.1 (22.7)	6.9
*NDRG1-OT1_v3*	0.0 (0.0)	0.0	4.0 (1.3)	7.7	0.0 (0.0)	0.0
*NDRG1-OT1_v4*	3.6 (42.3)	9.3	182.4 (58.0)	490.17	6.8 (74.3)	16.7
*NDRG1-OT1_v5*	0.4 (4.3)	7.6	8.2 (2.6)	175.17	0.3 (3.0)	5.5

FPKM, fragments per kilobase of transcript per million mapped reads.

### Gene expression profiling in MCF-7 cells overexpressing *NDRG1-OT1*_v4

**Figure 3:**
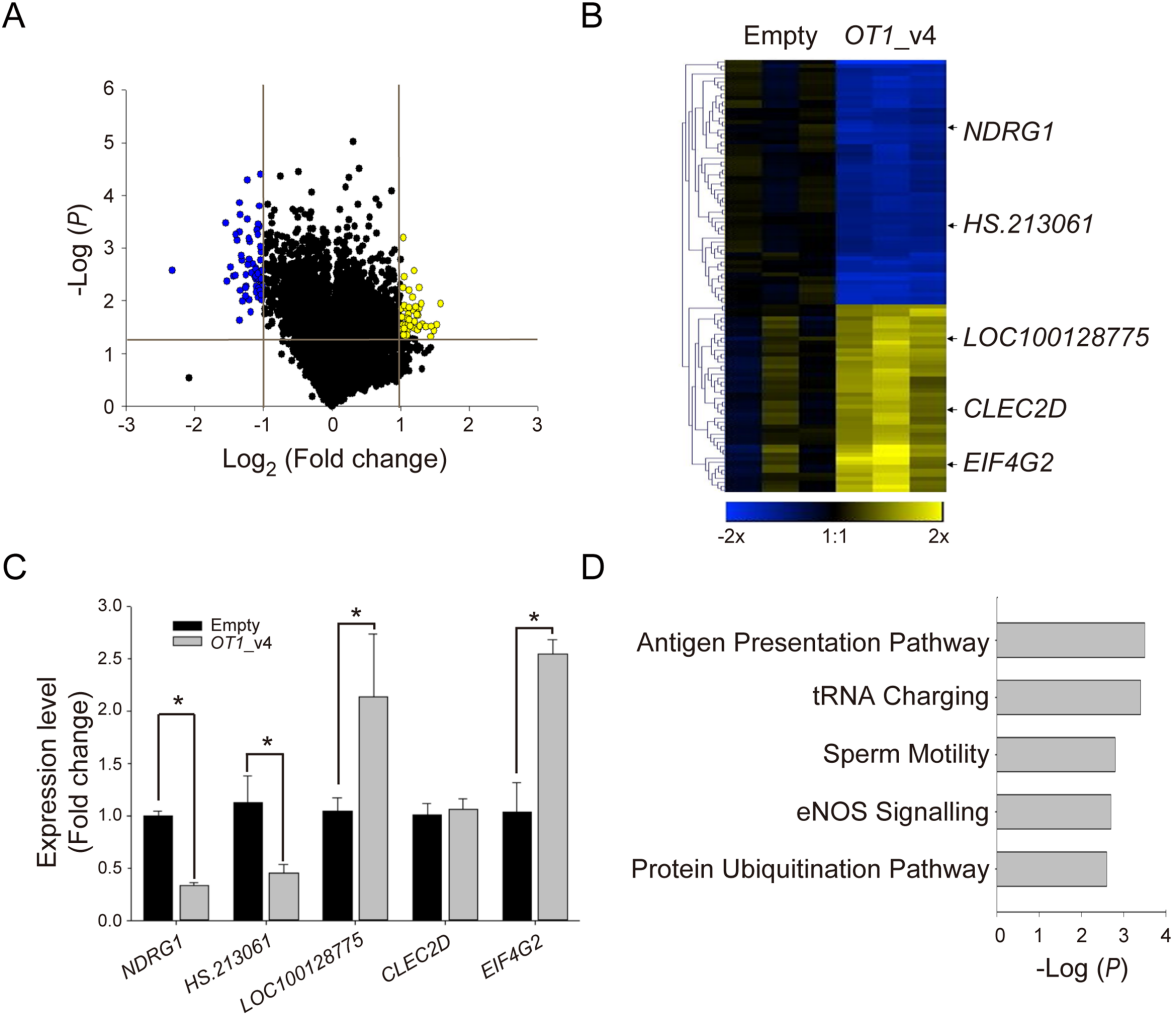
Identification of *NDRG1-OT1*_v4-regulated genes using microarray analysis **(A)** Volcano plot of differentially expressed genes in MCF-7 cells over-expressing *NDRG1-OT1*_v4 in O_2_. Criteria for selecting *NDRG1-OT1*_v4 regulated genes: fold change >2X and *P* <0.05. Yellow points: up-regulated genes in cells over-expressing *NDRG1-OT1*_v4; blue points: down-regulated genes. **(B)** Heat map and hierarchical cluster analysis of *NDRG1-OT1*_v4-regulated genes. Yellow: up-regulated genes in cells over-expressing *NDRG1-OT1*_v4; blue: down-regulated genes. Top five differentially expressed genes were indicated. **(C)** Relative transcriptional levels of the top five differentially expressed genes in MCF-7 cells overexpressing *NDRG1-OT1*_v4 by quantitative RT-PCR. Internal control: 18s. The relative expression level was normalized to empty vector. **(D)** Top five pathways in which *NDRG1-OT1*_v4 regulated genes were enriched using Ingenuity Pathway Analysis (IPA). The significance of pathways was determined by IPA's default threshold (−log(*P*-value) < 1.3).

In order to identify genes regulated by *NDRG1-OT1*_v4, the gene expression of wild type MCF-7 cells and MCF-7 cells overexpressing *NDRG1-OT1*_v4 in O_2_ was compared using Illumina Human HT-12 v4 BeadChips. Student's t-test was used to examine the difference in gene expression intensities. The criteria for defining *NDRG1-OT1*_v4-regulated genes included fold change > 2 and significant differences (*P* <0.05) (Figure 3A). In total, 108 genes met the criteria, with 47 induced by *NDRG1-OT1*_v4 and 61 inhibited by *NDRG1-OT1*_v4 (Figure 3B). To validate the *NDRG1-OT1*_v4-regulated genes identified by microarray, we used RT-PCR to examine the top five differentially expressed genes responding to ectopic *NDRG1-OT1*_v4 (Table 2). As shown in Figure 3C, the results of RT-PCR, except *CLEC2D*, were similar to those of microarray. Furthermore, pathway analysis showed that these genes were involved in the antigen presentation pathway, tRNA charging, sperm motility, eNOS signaling, and protein ubiquitination (Figure 3D).

**Table 2:** Top five differentially expressed genes responding to ectopic *NDRG1-OT1*_v4

Gene	Fold change (log_2_)	*P*-value	Rank (|Fold change|)
*NDRG1*	−2.32	3.0×10^−3*^	1
*HS.213061*	−1.58	1.2×10^−2*^	2
*LOC100128775*	1.53	4.0×10^−3*^	3
*EIF4G2*	1.53	2.9×10^−2*^	4
*CLEC2D*	1.49	3.7×10^−2*^	5

^*^P <0.05 was considered significant.

### *NDRG1* mRNA and protein levels are down-regulated by *NDRG1-OT1*_v4

**Figure 4:**
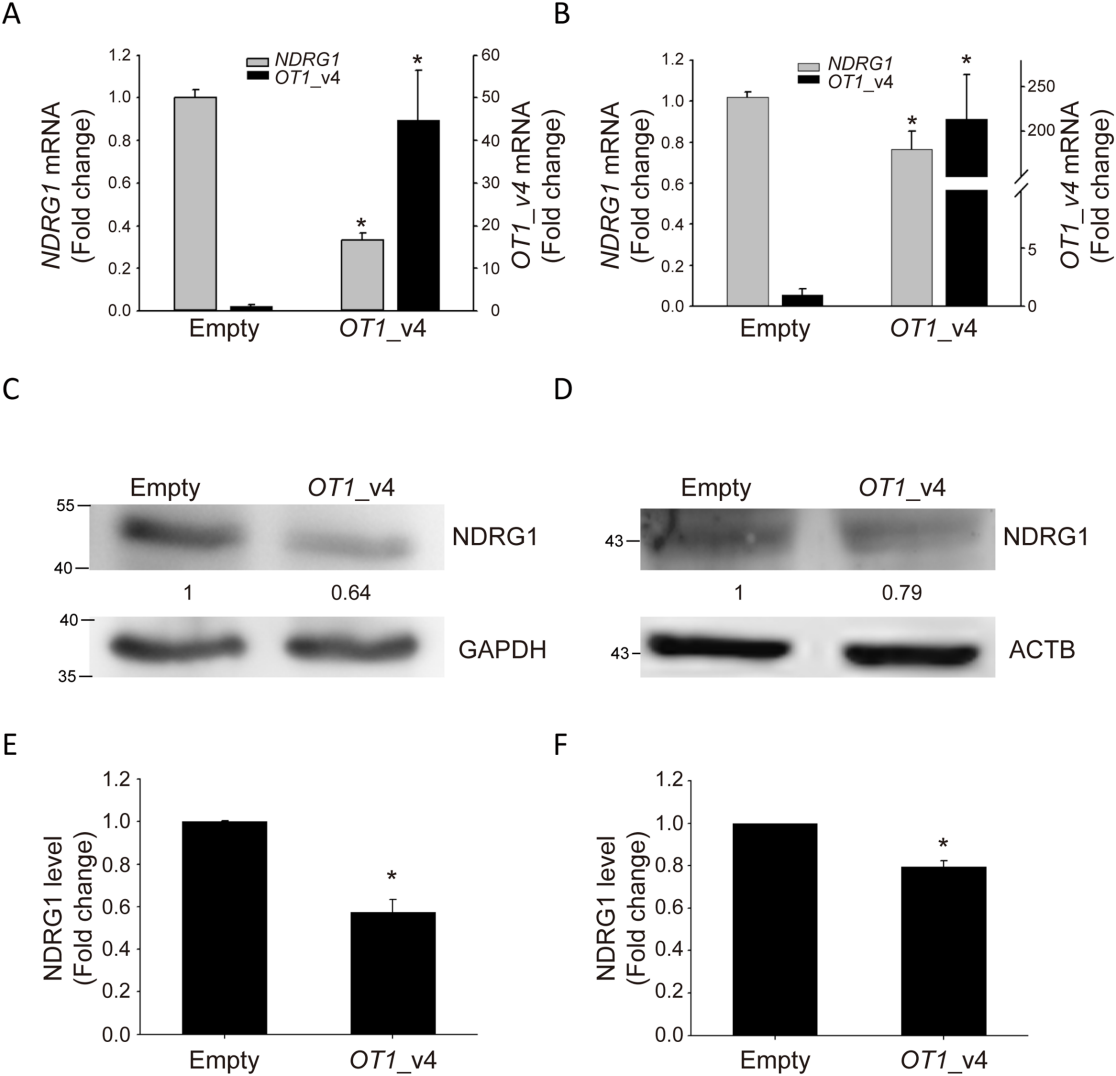
*NDRG1-OT1*_v4 inhibits *NDRG1* mRNA and protein levels **(A** and **B)** Relative transcriptional level of *NDRG1* (gray, left axis) and *NDRG1-OT1*_v4 (black, right axis) in MCF-7 (A) and SKBR3 (B) cells overexpressing *NDRG1-OT1*_v4. Two μg of plasmid were transfected into MCF-7 cells. *NDRG1* mRNA was measured by quantitative RT-PCR. Internal control: 18s. The relative expression levels were normalized to empty vector. **(C** and **D)** Western blot of NDRG1 in *NDRG1-OT1*_v4 overexpressed MCF-7 (C) and SKBR3 (D) cells. Relative protein amount of NDRG1 in MCF-7 cells overexpressing *NDRG1-OT1*_v4. Internal control: GAPDH (C) and ACTB (D). **(E** and **F)** Quantification of NDRG1 in MCF-7 (E) and SKBR3 (F) cells transfected with *NDRG1-OT1*_v4 plasmid. The relative expression levels were normalized to empty vector. Data were repeated at least 3 times, and the results are the means ± SDs ^*^, *P* <0.05.

Among the top five differentially expressed genes, we were particularly interested in *NDRG1* because it is a hypoxia-responsive gene with the highest fold change in response to ectopic expression of *NDRG1-OT1*_v4. We used quantitative RT-PCR and western blotting to validate *NDRG1* mRNA (Figure 4A&4B) and protein levels (Figure 4C–4F) in MCF-7 and SKBR3 breast cancer cells. Both transcript and protein levels of *NDRG1* were significantly down-regulated in cells overexpressing *NDRG1-OT1*_v4.

### *NDRG1_OT1*_v4 promotes NDRG1 ubiquitination and degradation

**Figure 5:**
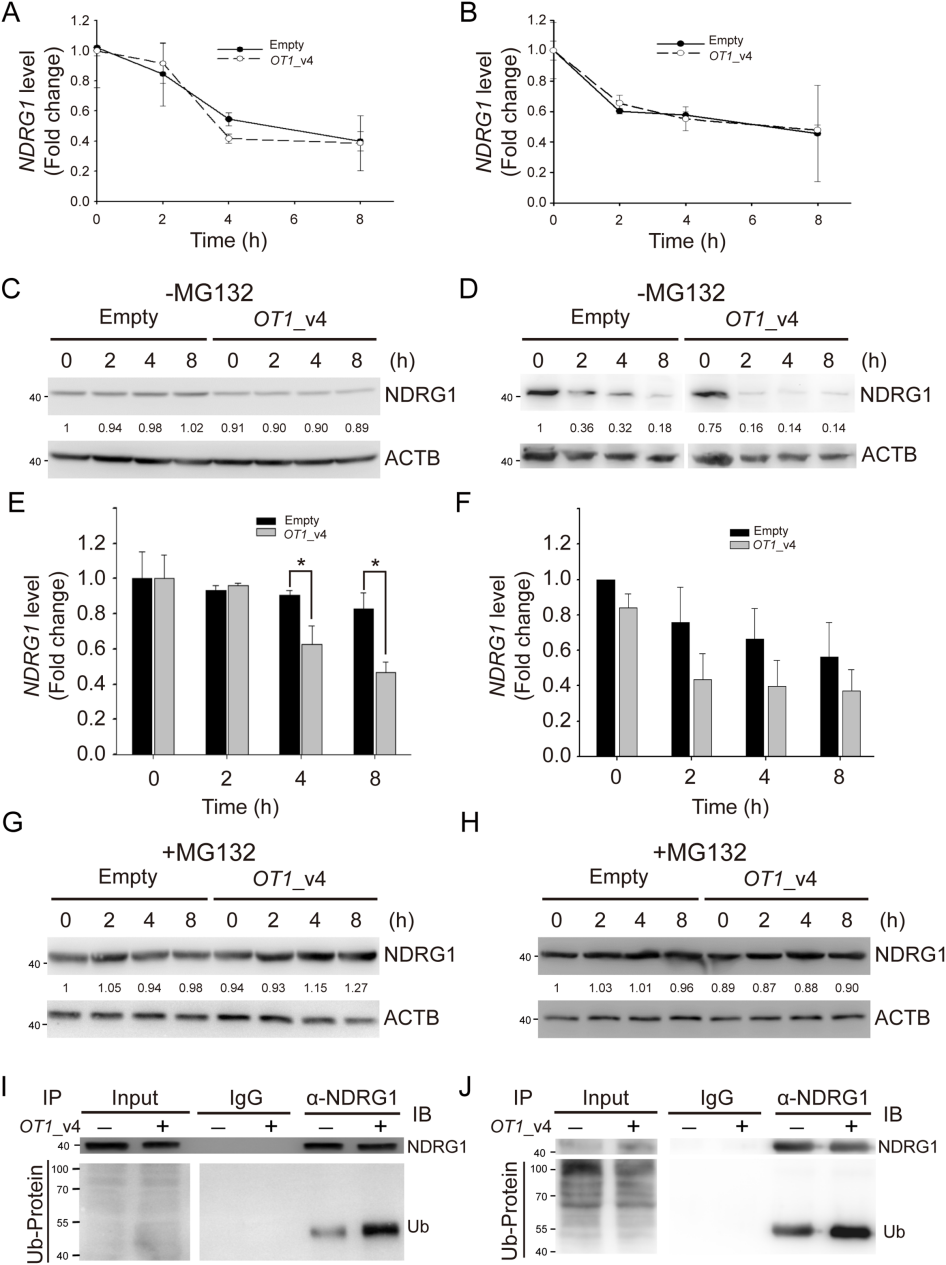
*NDRG1-OT1*_v4 promotes NDRG1 degradation via ubiquitination **(A** and **B)** Temporal profile of *NDRG1* in *NDRG1-OT1*_v4-overexpressed MCF-7 (A) and SKBR3 (B) cells treated with actinomycin D (10 μg/mL). *NDRG1* mRNA was measured by quantitative RT-PCR. Internal control: 18s. **(C** and **D)** Western blot of NDRG1 in *NDRG1-OT1*_v4-overexpressed MCF-7 (C) and SKBR3 (D) cells treated with cycloheximide (10 μg/mL). **(E** and **F)** Quantification of NDRG1 in (C&D). The results are the means ± SDs. ^*^, *P* <0.05. **(G** and **H)** Western blot of NDRG1 in *NDRG1-OT1*_v4-overexpressed MCF-7 (G) and SKBR3 (H) cells treated with cycloheximide (10 μg/mL) and MG132 (20 μg/mL). **(I** and **J)** Co-immunoprecipitation of NDRG1 and ubiquitin in MCF-7 (I) and SKBR3 (J) cells overexpressing *NDRG1-OT1*_v4 in the presence of MG132 (20 μg/mL). Cells were immunoprecipitated with NDRG1 antibody, followed by western blotting. Data were repeated at least 3 times.

Since the protein ubiquitination pathway was one of the pathways that *NDRG1-OT1_*v4-regulated genes were involved in (Figure 3D), we next investigated whether *NDRG1-OT1*_v4 down-regulated NDRG1 by affecting the stability of NDRG1. We first examined the stability of *NDRG1* mRNA by treating cells with a transcriptional inhibitor, actinomycin D (10 μg/mL). MCF-7 & SKBR3 cells were harvested at the indicated time points (0, 2, 4, 8 h) after treatment. As shown in Figure 5A&5B, the degradation of *NDRG1* mRNA was not affected by *NDRG1-OT1*_v4.

Next, in order to examine the effects of *NDRG1-OT1*_v4 on stability of NDRG1 protein, cells overexpressing *NDRG1-OT1*_v4 were examined by treating with a protein synthesis inhibitor, cycloheximide (10 μg/mL). Within 8 h after cycloheximide treatment, the protein levels of NDRG1 were significantly diminished in cells overexpressing *NDRG1-OT1*_v4 compared to control cells, indicating *NDRG1-OT1*_v4 promoted NDRG1 degradation (Figure 5C–5F). When MCF-7 & SKBR3 cells were treated with both cycloheximide and MG132, a proteasome inhibitor, *NDRG1-OT1*_v4 no longer promoted NDRG1 degradation (Figure 5G&5H). In addition, to examine whether down-regulation of NDRG1 by *NDRG1-OT1*_v4 was via the ubiquitin-proteasome system, we performed immunoprecipitation with an anti-NDRG1 antibody, followed by immunoblotting using an anti-ubiquitin antibody. The results showed that *NDRG1-OT1*_v4 increased ubiquitin binding to NDRG1 (Figure 5I&5J). These results suggest that *NDRG1-OT1*_v4 inhibits NDRG1 expression through the ubiquitin-proteasome pathway.

## DISCUSSION

To identify oxygen-responsive lncRNAs in breast cancer, we identified 472 lncRNAs responding to changes in oxygen concentration. Among these lncRNAs, *NDRG1-OT1*, especially *NDRG1-OT1*_v4 isoform, was the most responsive to oxygen changes. Next, *NDRG1-OT1*_v4 was overexpressed in MCF-7 cells under normoxia and *NDRG1* was chosen for further analysis because it had the greatest fold change in expression. Lastly, we identified the mechanism by which *NDRG1-OT1*_v4 inhibits NDRG1 via ubiquitin-mediated proteolysis.

In this study, NGS was used to identify oxygen-responsive lncRNAs in breast cancer MCF-7 cells (Figure 1). Among the oxygen-responsive lncRNAs, *NDRG1-OT1* was chosen for further analysis based on several reasons. First, *NDRG1-OT1* was in the top three lncRNAs that were strongly up-regulated under hypoxia and down-regulated under re-oxygenation. Second, since lncRNA usually up/down-regulates its neighboring genes [27, 35], we found that *NDRG1-OT1* was transcribed within the hypoxia-inducible gene *NDRG1* (Figure 2B). Therefore, we hypothesized that *NDRG1-OT1* may participate in breast cancers acclimatizing to hypoxia and hence chose *NDRG1-OT1* for further experiments.

lncRNAs usually have more than one transcript form, and different isoforms have different biological functions. For example, *UCA1* has three isoforms, but only the 1.4 kb (v1) isoform promotes bladder cancer proliferation and metastasis [31]. Similarly, *NDRG1-OT1* has five isoforms. Four of the five *NDRG1-OT1* isoforms, including v4, contained the *NDRG1* 3’-UTR, but only *NDRG1-OT1*_v4 contained parts of the *NDRG1* 14^th^ exon and 14^th^ intron. Since *NDRG1-OT1*_v4 had the highest expression level and proportion under hypoxia, we speculated that the 14^th^ exon and 14^th^ intron of *NDRG1-OT1*_v4 may play a pivotal role in regulating cell functions under hypoxia.

To explore the function of *NDRG1-OT1_*v4, we used microarray to identify genes regulated by *NDRG1-OT1_*v4. Pathway analyses showed that these *NDRG1-OT1_*v4 regulated genes were involved in the antigen presentation pathway, tRNA charging, sperm motility, endothelial nitric oxide synthase (eNOS) signaling, and the proteasome-ubiquitination pathway (Figure 3D). Since *NDRG1-OT1*_v4 was a hypoxia-inducible lncRNA, these pathways should be associated with hypoxia too. Indeed, previous studies have demonstrated that hypoxia increased T cell stimulation in dendrite cells [36], cardiac and hepatic eNOS production [37], and protein ubiquitination [38]. Hypoxia also negatively regulated the motility of sperm [39]. Moreover, some studies have discovered that hypoxia influences ubiquitin-mediated proteolysis via lncRNAs [23, 32].

In this study, we found that *NDRG1-OT1*_v4 inhibited its overlapped gene *NDRG1* at both mRNA and protein levels in at least two breast cancer cell lines, MCF-7 & SKBR3 (Figure 4). According to previous findings, expression levels of several lncRNAs were mainly correlated with those of their nearest genes, resulting in either a positive [27] or negative effect [26]. Although our findings indicated that *NDRG1-OT1*_v4 had a negative effect on *NDRG1*, *NDRG1-OT1*_v4 does not seem to play a major role. Previous studies have demonstrated that *NDRG1* expression under different oxygen concentrations was regulated by both transcriptional [14, 40] and epigenetic factors [17]. Therefore, *NDRG1-OT1*_v4 is likely one of several regulatory mechanisms that fine tunes the expression of *NDRG1* in response to changes in oxygen concentrations.

In addition, mRNA and protein levels of *NDRG1* were both decreased by overexpressing *NDRG1-OT1*_v4. Regarding mRNA stability, upon treatment with the transcriptional inhibitor actinomycin D, we found that *NDRG1-OT1*_v4 did not affect *NDRG1* mRNA stability (Figure 5A & 5B). Unlike other lncRNA, such as *TINCR*, it has been found to regulate mRNA stability [28]. Therefore, we expected that *NDRG1-OT1*_v4 might inhibit the transcription of *NDRG1*. Deletion analysis of lncRNA followed by luciferase reporter assays or dot-blot assays [41] could be used to identify the pivotal region of lncRNA regulating its target genes.

On the other hand, when MCF-7 cells were treated with the protein synthesis inhibitor cycloheximide, *NDRG1-OT1*_v4 promoted NDRG1 degradation via ubiquitin-mediated proteolysis (Figure 5C–5J). Although previous studies have indicated that lncRNAs can promote protein stability by blocking protein from interacting with ubiquitin [19, 23, 32], we observed an opposite effect of lncRNA on ubiquitin-mediated proteolysis. Namely, we first reported that *NDRG1-OT1*_v4 destabilized NDRG1 by promoting ubiquitination (Figure 5C–5J).

There were some limitations in this study. First, in order to investigate *NDRG1-OT1*_v4-regulated genes, using both overexpression and silencing of *NDRG1-OT1*_v4 would be optimal to reduce the false positives. However, when we tried to knock down *NDRG1-OT1*_v4 under hypoxia by transducing several shRNAs against *NDRG1-OT1*_v4, not only was the *NDRG1-OT1*_v4 down-regulated but also its downstream gene *NDRG1*. Because of this off-target effect, we only conducted the experiments on ectopically expressed *NDRG1-OT1*_v4. Second, to examine whether this novel regulatory mechanism of *NDRG1-OT1*_v4 exists in other cancer types, similar experiments should be performed in different cancer cell lines and clinical samples.

In conclusion, we identified a group of oxygen-responsive lncRNAs in breast cancer cells and a novel regulatory mechanism of *NDRG1-OT1*_v4 by enhancing the degradation of its down-stream target NDRG1 protein.

## MATERIALS AND METHODS

### Cell culture and treatments

Breast cancer cell lines, MCF-7, SKBR3, and MDAMB-231, were maintained in Dulbecco's modified Eagle's medium (DMEM) (GIBCO, Carlsbad, USA), which was supplemented with 10% fetal bovine serum (FBS) (GIBCO) and 1% penicillin-streptomycin-amphotericin solution (Biological Industries, Beit-Haemek, Israel). Breast cancer cell line ZR75-30 was maintained in Roswell Park Memorial Institute (RPMI). Breast cancer cell lines, MDAMB-361 and MDAMB-453, were cultured in Leibovitz's L-15 (L15) culture medium (GIBCO) with 20% FBS and 1% antibiotics. Normal cell line MCF-10A were maintained in DMEM and Ham's Nutrient Mixture F12 (F12) mixed buffer (GIBCO, Carlsbad, CA, USA) with 5% horse serum, 20 ng epidermal growth factor, 0.5 mg hydrocortisone, 100 ng cholera toxin, 10 μg insulin, and 1% penicillin-streptomycin-amphotericin solution (Biological Industries, Beit-Haemek, Israel).

Except MDAMB-361 and MDAMB-453 growing in a humidified atmosphere without CO_2_, other cells were incubated at 37°C in a humidified atmosphere with 5% CO_2_, or in an *In VivO* 2-200 hypoxia chamber (Ruskinn Technology, Leeds, UK) filled with a gas mixture of 0.5% O_2_, 5% CO_2_ and 94.5% N_2_, for 24 h. For the drug treatment experiment, MCF-7 cells were mixed with 10 μg/mL cycloheximide (Merck, Darmstadt, Germany) or 10 μg/mL actinomycin D (Sigma, St. Louis, USA).

### Next-generation sequencing and analysis of oxygen-responsive lncRNA

Total RNA extracted from MCF-7 was digested to 180 bp fragments after removing rRNAs with a Ribo-Zero Gold kit (Illumina, San Diego, USA). The RNA fragments were reverse-transcribed to cDNA using a TruSeq Stranded total RNA kit (Illumina), and were ligated to specific adapters at both ends of these cDNA fragments to produce a sequencing library. Next, the sequencing libraries were hybridized with adapter's oligonucleotide sequence on the surface of flow cells (Illumina). Each bound fragment was amplified into a clonal cluster through bridge amplification. All of the clusters on the flow cell were finally sequenced by HiScan SQ (Illumina).

The FastQC program (http://www.bioinformatics.babraham.ac.uk/projects/fastqc/) was used to examine the quality control of sequencing reads, and TopHat 2 (https://ccb.jhu.edu/software/tophat/indshex.tml) was used to align sequencing reads to human genome references (GRCh37/hg19). Then, these mapped reads were matched to lncRNAs from the Lncipedia database (http://www.lnciedia.org/) and were normalized to Fragments Per Kilobase of transcript per Million mapped reads (FPKM) as the expression level of a given lncRNA using Cufflink (http://cole-trapnell-lab.github.io/cufflinks/). Finally, Cuffdiff (http://cole-trapnell-lab.github.io/cufflinks/cuffdiff/) was utilized to compare the different FPKMs for each condition. All of the data have been deposited in Gene Expression Omnibus (GEO, GSE84167).

### Plasmid DNA construction and transfection

The pUC57 plasmid containing *NDRG1-OT1*_v4 was purchased from GenScript (GenScript, Piscataway, USA), digested by *Bam*H1 (BioLabs, New England, UK) and *Eco*R1 (BioLabs), and inserted into the pcDNA3.1^+^ plasmid (Thermo Fisher Scientific Taiwan). MCF-7 cells were transfected with pcDNA3.1^+^-*NDRG1-OT1*_v4 or empty vector using jetPRIME transfection reagent (Polyplus-transfection, New York, USA) according to the manufacturer's instructions. After 4 h, the transfection medium was replaced with fresh medium-containing serum. After 24 h, cells were checked for RNA expression by quantitative RT-PCR.

### RNA extraction and quantitative RT-PCR

Total RNA was extracted by TRIpure reagent (Roche Diagnostics, Branchburg, USA) according to the manufacturer's protocol. One μg of total RNA was reverse-transcribed to cDNA using a High-Capacity cDNA Reverse Transcription Kit (Applied Biosystems, Carlsbad, USA). Ten percent of each cDNA was used as the template for quantitative real-time PCR with FastStart Universal SYBR Green Master reaction mixture (Roche) and custom primers (Table 3). Finally, the reaction was carried out on an ABI Step One plus system (Applied Biosystems). Each reaction was done in triplicate, and data were normalized to loading control 18s rRNA.

**Table 3:** The primers for quantitative RT-PCR

Gene/lncRNA	Sequence (5’ to 3’)
*NDRG1-OT1_v4*	(F) CTCCCAGGTTCCTGTACTACTG (R) GGCGGCAGGTAACGAGTCATTG
*NDRG1*	(F) GGCAACCTGCACCTGTTCATCAAT (R) TGAGGAGAGTGGTCTTTGTTGGGT
18s rRNA	(F) TCAACTTTCGATGGTAGTCGCCGT (R) TCCTTGGATGTGGTAGCCGTTTCT

F, forward; R, reverse.

### Human genome microarray analysis

The total RNA was primed with T7 Oligo(dT) and amplified by an Illumina TotalPre RNA Amplification Kit (Ambion, Austin, USA) to synthesize the cDNA containing a T7 promoter sequence. Following the first strand of cDNA synthesis, the second strand of cDNA was synthesized by converting the single-stranded cDNA into a double-stranded DNA (dsDNA) template for transcription. The reaction employed DNA polymerase and RNAase H to simultaneously degrade the RNA and synthesize the second strand of cDNA. After cleanup, *in vitro* transcription was conducted using the double-stranded cDNA as a template and T7 RNA polymerase to synthesize multiple copies of biotinylated complementary RNA (cRNA). After amplification, the cRNA was mixed with an equal volume of hybridization buffer and hybridized to Illumina Human HT-12 v4 BeadChips (Illumina) at 58°C for 16 h. After hybridization, the BeadChip was washed and stained with streptavidin-Cy3 dye. The intensity of the beads’ fluorescence was detected by HiScan SQ (Illumina), and the results were analyzed using BeadStudio software.

After scanning, the intensity data of Illumina Human HT-12 v4 BeadChips were analyzed using Partek software (Partek, St. Charles, USA) for mRNA analysis. Background-adjusted signals were normalized by a quantile normalization algorithm. All data have been uploaded to GEO (GSE72881). Furthermore, Ingenuity Pathway Analysis (Ingenuity Systems Inc. Redwood City, USA) was applied to comprehend the biological functions and signaling pathways of differentially expressed genes.

### Protein extraction and western blotting

MCF-7 cells were lysed in RIPA lysis buffer (Sigma), and protein concentration was determined using Bio-Rad Protein assay reagent (Bio-Rad Laboratories, Hercules, USA). Then, the protein lysate was separated by 10% SDS-PAGE and transferred to a PVDF membrane (Bio-Rad Laboratories). The membranes were blocked by TBST Blocking buffer (Arrowtec, Taiwan) for 10 min and hybridized to a primary antibody consisting of NDRG1 (Abcam Inc., Cambridge, USA) and ubiquitin (Abcam). After immunoblotting, the membranes were washed by TBS (Omics Bio, Taiwan) with Tween20 and reacted with horseradish peroxidase-conjugated goat anti-rabbit IgG or rabbit anti-mouse IgG (GeneTex, Irvine, USA). The protein bands were visualized using an enhanced chemiluminescence system (Millipore, Billerica, USA).

### Co-immunoprecipitation

The immunoprecipitation antibodies, 5 μg NDRG1 Ab (Abcam) and 5 μg normal mouse IgG (Millipore), were reacted with 50 μL G beads (Life Technology, Gaithersburg, USA) in 200 μL Ab binding & washing buffer (Life Technology), followed by overnight shaking at 4°C. MCF-7 cells were harvested and lysed in RIPA buffer (Sigma) after reacting with 20 μM MG132 (Abmol, Houston, USA) for 4 h. Then, protein concentration was determined using Bio-Rad Protein assay reagent (Bio-Rad Laboratories). One thousand μg of protein lysate were reacted with Ab-G beads during overnight shaking at 4°C. After immunoprecipitation, protein-Ab-G bead complexes were separated and washed with washing buffer (Life Technology). Finally, protein-Ab-G bead complexes were boiled for 10 min, and western blotting was performed.

### Statistical analysis

All data are reported as means ± SDs for at least 3 independent experiments. The difference between each group was analyzed using Student's t test. *P* <0.05 was considered significant, if no other α value was specified.
